# Association between the Balanced Healthy Eating Index and depression and the mediating role of extreme pessimistic thoughts: an analysis from the health and nutrition database

**DOI:** 10.3389/fnut.2025.1590171

**Published:** 2025-08-19

**Authors:** Yantong Liu, Shengyong Bao, Chuang Li, Feifei Li, Mingqian Liang, Guang Yang

**Affiliations:** ^1^Department of Neurology, Kunshan Hospital of Traditional Chinese Medicine, Kunshan, Jiangsu, China; ^2^Department of Computer and Information Engineering, Kunsan National University, Gunsan, Republic of Korea; ^3^Department of Rehabilitation Medicine, Shenzhen People’s Hospital (The Second Clinical Medical College, Jinan University, The First Affiliated Hospital, Southern University of Science and Technology), Shenzhen, China; ^4^Department of Biological Sciences, Purdue University, West Lafayette, IN, United States; ^5^Molecular Pharmacology and Experimental Therapeutics, Mayo Clinic, Rochester, MN, United States

**Keywords:** Balanced Healthy Eating Index, extreme pessimism, depression, NHANES, diet quality

## Abstract

**Background:**

Depression remains a significant global health issue, yet the combined influence of dietary quality and extreme pessimistic thoughts on depressive risk is not fully understood. This study evaluated whether a Balanced Healthy Eating Index (BHEI) and extreme pessimism independently and jointly predict depression in adults.

**Materials and methods:**

We analyzed data from 17,575 participants aged 18–65 years in the 2007–2018 National Health and Nutrition Examination Survey (NHANES). BHEI scores above 62 were classified as meeting healthy dietary standards. Logistic regression models assessed the associations between substandard BHEI, extreme pessimism (frequent thoughts of being better off dead), and self-reported depression (PHQ-9 ≥ 10). Models were adjusted for age, sex, ethnicity, BMI, total energy intake, smoking status, alcohol use, and physical activity.

**Results:**

Participants failing to meet the BHEI threshold had increased odds of depression (OR = 1.55, 95% CI: 1.37–1.76). Extreme pessimism further elevated depression risk (OR = 4.17, 95% CI: 3.40–4.41). An interaction effect showed that individuals with both substandard BHEI and extreme pessimism faced even higher odds of depression (OR = 8.05, 95% CI: 6.62–9.07), suggesting a multiplicative relationship.

**Conclusion:**

Both low-quality diets and extreme pessimistic thinking were significantly associated with depression risk, and the combination was particularly impactful. Future longitudinal studies are warranted to clarify causal pathways and to determine whether improving dietary patterns or mitigating extreme pessimism could reduce depression prevalence.

## 1 Introduction

Depressive disorders pose a substantial global health burden, affecting people across populations and contributing to increased morbidity, mortality, and economic costs (1, 2). According to existing epidemiological evidence, depression prevalence varies notably by demographic characteristics such as age, gender, socioeconomic status, and chronic comorbidities (3, 4). Gender is frequently reported as a determinant, with women exhibiting higher rates of depression than men, potentially due to hormonal fluctuations and psychosocial factors (5). Genetic predispositions, indicated by the familial aggregation of depressive disorders, further increase susceptibility (6). Additionally, modifiable lifestyle behaviors, including tobacco use, alcohol consumption, and physical inactivity, have been consistently linked to higher depression risk (7, 8). Chronic health conditions, such as cardiovascular disease and diabetes, also elevate the risk of depressive symptoms and complicate treatment outcomes (9, 10). Moreover, socioeconomic variables, such as marital status, income, education level, and stressful life events, significantly influence depressive symptomatology (11–14). Given these multifactorial influences, there is growing interest in the role nutritional factors play in the onset and progression of depressive symptoms.

Dietary factors have emerged as potential preventive and adjunct therapeutic targets for depressive disorders. Emerging research suggests that nutritional intake can influence mental health through pathways such as inflammation, oxidative stress, and gut–brain axis interactions (15, 16). Evaluations of diet quality and its association with mental health have utilized various dietary indices, among which the Healthy Eating Index (HEI), including the Healthy Eating Index, stand out due to their broad application in assessing dietary guideline adherence (17). These indices summarize multiple dietary components—such as fruit, vegetables, whole grains, saturated fat, and added sugars—into composite scores to provide a holistic assessment of dietary quality (17, 18). Prior research linking dietary patterns assessed by HEI to depression revealed mixed findings. Some studies report inverse associations, suggesting that healthy dietary patterns are protective against depression (19–23). However, the mechanisms by which dietary, behavioral, and sociodemographic determinants play a role in influencing mental health outcomes remain complex (24, 25). Thus, further research leveraging robust population-based data are warranted to better delineate how specific dietary components may impact depression risk.

Against this background, the present study aims to investigate the association between different forms of the Healthy Eating Index (HEI) and depression among adults aged 18 and 65 years, drawing data from the 2007–2018 National Health and Nutrition Examination Survey (NHANES) (26). Additionally, we seek to explore the potential mediating role of extreme pessimistic thoughts—conceptualized here as instances where respondents frequently consider that they might be “Thought you would be better off dead” on the link between diet quality and depressive symptomatology (27). By leveraging the comprehensive scope of NHANES, which includes detailed dietary intake data, demographic information, and mental health assessments, we aim to illuminate whether specific dimensions of healthy eating can forecast reduced susceptibility to depression and whether extreme pessimistic thinking independently mediates that relationship (28). This study seeks to establish whether adherence to a healthy diet is associated with reduced depressive symptoms and whether extreme pessimistic thoughts mediate this association, thereby highlighting potential cognitive and behavioral intervention points.

## 2 Materials and methods

### 2.1 Data sources

NHANES is a periodic cross-sectional survey designed to assess the health status of the US population. It is conducted every 2 years and uses a multistage probability sampling design to ensure a nationally representative sample from different geographical regions of the United States. The database consistently assesses depression using the 9-item Mental Health Questionnaire (PHQ-9). Due to the disruption caused by the COVID-19 pandemic, the scarcity and complexity of data collected after 2019 may not accurately reflect the prevalence of depression in the current population. Therefore, to ensure data consistency and reliability, the NHANES cycle from 2007 to 2018 was selected for our analyses (29).

### 2.2 Research participants

Participants aged between 18 and 65 years who underwent dietary survey data, as well as psychological questionnaires, were included in this study. Subjects with incomplete demographic, dietary, or questionnaire data were excluded; those indexed to the abnormal energy intake criteria were excluded; and those with other dietary abnormalities were excluded. A total of 17,575 subjects met these criteria and were included in the analysis, as detailed in Table 1.

**Table 1:** Overall baseline characteristics of general demographic data (*n* = 17,575).

Variable	Number of cases/weighted median	Weighted percentage (%)/(P25.P75)
Sex		
Male	9,033	51.18
Female	8,542	48.82
Age/year	40	−30.52
Ethnicity		
Mexican American	1,576	8.97
Non-Hispanic White	12,164	69.21
Non-Hispanic Black	1970	11.21
Other race	1865	10.61
Total energy intake/kcal	2037	(1,509, 2,707)
BMI		
< 25.0	5,258	29.92
≥ 25.0 and < 30.0	5,609	31.91
≥ 30.0	6,708	38.17
Smoking status		
Smoking	7,961	45.30
Non-smoking	9,614	54.70
Alcohol consumption		
Mild	7,735	44.01
Moderate	4,255	24.21
Severe	5,585	31.78
Physical activity		
Yes	7,914	45.03
No	9,661	54.97
Depressive conditions		
Yes	1,589	9.04
No	15,986	90.96
Extreme pessimistic thoughts		
Yes	676	3.84
No	16,899	96.16

### 2.3 Depression criteria

Depressive symptoms are the outcome measure. The PHQ-9 depression screening scale consists of 9 items, each of which is scored on a 4-point scale of 0, 1, 2, and 3, for a total score of 27, with a score of 0 to 9 indicating no depression and a score of 10 to 27 indicating the presence of depression (30). Depressive symptoms were measured using the Patient Health Questionnaire-9 (PHQ-9), a validated self-report instrument commonly applied to both clinical and population-based studies. In NHANES, this questionnaire is administered via the Audio Computer-Assisted Self-Interview (ACASI) system at the Mobile Examination Center. Each item is scored from 0 (not at all) to 3 (nearly every day), with a total score ranging from 0 to 27. Extreme pessimistic thoughts were assessed using item 9 of the PHQ-9, which inquires about thoughts of death or self-harm.

Extreme pessimism was defined based on item 9 of the PHQ-9, which captures passive or active thoughts of death or self-harm. While this item is often used as a proxy for hopelessness in depression research, it reflects only the most severe end of pessimistic thinking.

### 2.4 Extreme pessimistic tendency criterion

To assess the presence of extreme pessimistic thoughts, participants were scored on their responses to item DPQ090 from the PHQ-9 questionnaire, which specifically asked the following question: “Over the last 2 weeks, how often have you been bothered by the following problems: Thoughts that you would be better off dead or of hurting yourself in some way?” Responses were categorized into four frequencies: 0 (not at all), 1 (several days), 2 (more than half of the days), and 3 (almost every day). Participants who answered ‘refused’ or ‘do not know’ were excluded from the analysis. For this study, individuals who selected responses coded as 1, 2, or 3 were classified as having suicidal ideation.

### 2.5 Balanced Healthy Eating Index (BHEI)

In this study, we used the standard Healthy Eating Index (HEI) as an established measure of dietary quality, and building on this approach, we introduced a Balanced Healthy Eating Index (BHEI) framework that incorporates some scaling adjustments to provide a refined assessment perspective by reweighting specific components. To enhance its specificity for depression-related outcomes, we retained the original 13 components of the HEI-2015 but adjusted their relative weights based on existing literature and exploratory analyses. The final BHEI score was normalized to 100 to ensure comparability across participants.

The dietary data used to construct the BHEI were derived from two 24-h dietary recall interviews conducted as part of NHANES. Nutrient and food group information were obtained from the dietary component files. The BHEI was calculated following previously established scoring protocols developed for NHANES-based diet quality assessment. For this analysis, participants with BHEI scores above 62 were considered to have reached a threshold for healthy eating.

### 2.6 Covariates

Covariates, include age, sex, race (Mexican American, non-Hispanic White, non-Hispanic Black, other), BMI (non-overweight BMI < 25.00 kg/m^2^, overweight 25.00 ≤ BMI < 30.00 kg/m^2^, and obese BMI ≥ 30.00 kg/m^2^), smoking history (more than 100 cigarettes, less than 100 cigarettes, or never smoked), alcohol consumption (heavy, moderate, and light), and physical activity (moderate to no activity).

### 2.7 Statistical analysis

Data were analyzed using IBM SPSS 27.0 and R 4.4.2. In accordance with NHANES analytical guidelines, data were weighted using the wtmec2yr variable to account for the complex survey design and 2-year sampling cycles.

Logistic regression models were used to examine the associations between the Balanced Healthy Eating Index (BHEI), pessimism, and depression. Interaction effects were also tested to assess the joint relationship between dietary quality and pessimism.

Three hierarchical models were constructed: Model 1 was adjusted for demographic variables (age, gender, race/ethnicity, education, and income); Model 2 was additionally adjusted for lifestyle factors (e.g., smoking, alcohol use, and BMI); and Model 3 further included psychological variables (e.g., sleep quality and antidepressant use).

For group comparisons, chi-square tests were applied to categorical variables. For continuous variables, normality was tested using the Shapiro–Wilk test. As most continuous variables were not normally distributed, the non-parametric Mann–Whitney U-test was used.

The performance of logistic regression models was assessed using the Hosmer–Lemeshow goodness-of-fit test and Nagelkerke pseudo R^2^ to evaluate model calibration and explanatory power.

## 3 Results

Baseline demographic characteristics are presented in Table 1. Group-wise distributions of depression-related factors are summarized in Table 2.

**Table 2:** Baseline characteristics of NHANES participants from 2007 to 2018 (*n* = 17,575).

Variables	Depressed group (*n* = 1,589)	Non-depressed group (*n* = 15,986)	Z/χ^2^	*p*
Sex/case (%)			112.263	< 0.001
Male	601(37.82)	8,432(52.75)		
Female	988(62.18)	7,554(47.25)		
Age/year	40(30.51)	41(29.52)	2.846	0.004
Ethnicity/case (%)			12.043	0.007
Mexican American	127(7.99)	1,449(9.06)		
Non-Hispanic White	1,066(67.08)	11,098(69.43)		
Non-Hispanic Black	179(11.26)	1791(11.20)		
Other race	217(13.67)	1,648(10.31)		
Extreme pessimism/case (%)			1195.628	< 0.001
Yes	596(37.51)	80(0.50)		
No	993(62.49)	15,906(99.50)		
Total energy intake/kcal	1921(1398.2537)	2053(1518.2717)	5.127	< 0.001
Balanced Healthy Eating Index/case (%)			84.927	< 0.001
BHEI score met	419(26.37)	5,866(36.69)		
BHEI not met	1,170(73.63)	10,120(63.30)		
BMI/case (%)			85.605	< 0.001
< 25.0 kg/m^2^	408(25.68)	4,850(30.34)		
≥25.0 and < 30.0 kg/m^2^	421(26.49)	5,188(32.45)		
≥30.0 kg/m^2^	760(47.83)	5,948(37.21)		
Smoking status/case (%)			240.702	< 0.001
Smoking	1,006(63.31)	6,955(43.51)		
Non-smoking	583(36.69)	9,031(56.49)		
Alcohol consumption/case (%)			8.831	< 0.001
Mild	572(36.00)	7,163(44.81)		
Moderate	408(25.68)	3,842(24.03)		
Severe	609(38.32)	4,976(31.16)		
Physical activity/case (%)			6.656	0.01
Yes	721(45.37)	7,193(44.99)		
No	868(54.63)	8,793(55.01)		

Statistical tests: chi-square for categorical variables; Mann–Whitney U-test for continuous variables.

### 3.1 Participant characteristics

A total of 17,575 subjects were included in this study, predominantly non-Hispanic White, with a majority of participants being non-smokers and light drinkers, a high proportion of people with a BMI above the normal range, and a high proportion of people who did not meet the BHEI. The distribution of general demographic characteristics is shown in Table 1.

The distribution of depression characteristics among NHANES study participants from 2007 to 2018 is shown in Table 2.

### 3.2 Mutual relationships

#### 3.2.1 Relationship between the BHEI index and depression

Logistic regression models were utilized to investigate the association between dietary inflammation and depression, using the non-depressed group as the reference. The corresponding results are summarized in Table 3. In all three models, dietary inflammation was significantly and positively associated with depression (*p* < 0.001). In Model 1, participants in the BHEI non-adherent group had a significantly higher risk of depression compared to the anti-inflammatory dietary group (OR = 1.60, 95% CI = 1.50–1.87, *p* < 0.001). In Model 1, participants in the BHEI non-adherent group had a significantly higher risk of depression compared to the anti-inflammatory dietary group (OR = 1.60, 95% CI = 1.50–1.87, *p* < 0.001). In Model 2, after adjusting for age, gender, and ethnicity, the risk of depression remained significantly elevated in the BHEI non-adherent group (OR = 1.58, 95% CI = 1.31–1.77, *p* < 0.001). In Model 3, after adjusting for age, gender, ethnicity, BMI, total energy intake, alcohol consumption, smoking status, and physical activity, participants in the BHEI non-adherent group still exhibited a significantly increased risk of depression (OR = 1.55, 95% CI = 1.37–1.76, *p* < 0.001).

**Table 3:** Logistic regression modelling of the Balanced Healthy Eating Index and depression.

Variable	Model 1		Model 2		Model 3	
	OR(95%CI)	*p*	OR(95%CI)	*p*	OR(95%CI)	*p*
Sex						
Male	–	–	1	–	1	–
Female	–	–	1.63(1.45 ~ 1.77)	< 0.001	1.84(1.65 ~ 2.05)	< 0.001
Age	–	–	1(1.00 ~ 1.01)	0.01	1.01(1.00 ~ 1.01)	0.07
Ethnicity						
Mexican American	–	–	1	–	1	–
Non-Hispanic White	–	–	1.17(1.01 ~ 1.37)	0.067	1.31(1.10 ~ 1.56)	0.012
Non-Hispanic Black	–	–	1.23(1.02 ~ 1.43)	0.123	1.21(1.05 ~ 1.47)	0.28
Other race	–	–	1.26(1.05 ~ 1.48)	0.12	1.31(1.10 ~ 1.56)	0.002
Balanced Healthy Eating Index						
BHEI score met	1	–	1	–	1	–
BHEI not met	1.60(1.50 ~ 1.87)	< 0.001	1.58(1.39 ~ 1.74)	< 0.001	1.55(1.37 ~ 1.76)	< 0.001
BMI	–	–	–	–	0.10(0.10 ~ 1.00)	0.287
Total energy intake	–	–	–	–	0.99(0.98 ~ 1.00)	0.12
Smoking status						
Smoking	–	–	–	–	2.33(1.96 ~ 2.43)	< 0.001
Non-smoking	–	–	–	–	1	–
Alcohol consumption						
Light	–	–	–	–	1	–
Moderate	–	–	–	–	1.05(0.96 ~ 1.25)	0.23
Heavy	–	–	–	–	1.49(1.30 ~ 1.66)	< 0.001
Physical activity						
Yes	–	–	–	–	1	–
No	–	–	–	–	1.11(1.01 ~ 1.24)	0.022

Model fit: Hosmer–Lemeshow test *p* = 0.523; Nagelkerke *R*^2^ = 0.22.

Model 1: no adjustment for other covariates; Model 2: adjusted for age, sex, and ethnicity; Model 3: adjusted for all covariates. *p*-values are based on Wald chi-square statistics from logistic regression.

#### 3.2.2 Relationship between extreme pessimistic thoughts and depression

Logistic regression models were used to investigate the association between extreme pessimism and depression. The results are presented in Table 4. Compared to the non-depressed population, participants with extreme pessimism had a significantly increased risk of depression across Models 1, 2, and 3 (*p* < 0.001).

**Table 4:** Logistic regression modelling of extreme pessimistic thoughts and depression.

Variable	Model 1		Model 2		Model 3	
	OR(95%CI)	*p*	OR(95%CI)	*p*	OR(95%CI)	*p*
Sex						
Male	–	–	1	–	1	–
Female	–	–	1.55(1.45 ~ 1.77)	< 0.001	1.56(1.65 ~ 2.05)	< 0.001
Age	–	–	1(1.00 ~ 1.01)	0.011	1.01(1.00 ~ 1.01)	0.07
Ethnicity						
Mexican American	–	–	1	–	1	–
Non-Hispanic White	–	–	0.88(0.78 ~ 1.07)	0.202	0.89(0.74 ~ 1.02)	0.115
Non-Hispanic Black	–	–	1.17(0.91 ~ 1.28)	0.312	1.14(0.95 ~ 1.35)	0.159
Other race	–	–	1.11(0.93 ~ 1.32)	0.245	1.15(0.96 ~ 1.37)	0.122
Extreme pessimistic thoughts						
No	1	–	1	–	1	–
Yes	4.41(3.57 ~ 4.67)	< 0.001	4.28(3.61 ~ 4.73)	< 0.001	4.17(3.40 ~ 4.41)	< 0.001
BMI	–	–	–	–	0.99(0.99 ~ 1.00)	0.077
Total energy intake	–	–	–	–	1.00(1.00 ~ 1.00)	0.287
Smoking status						
Smoking	–	–	–	–	2.01(1.75 ~ 2.18)	< 0.001
Non-smoking	–	–	–	–	1	–
Alcohol consumption						
Light	–	–	–	–	1	-
Moderate	–	–	–	–	1.10(0.96 ~ 1.25)	0.177
Heavy	–	–	–	–	1.47(1.30 ~ 1.66)	< 0.001
Physical activity						
Yes	–	–	–	–	1	–
No	–	–	–	–	1.12(1.01 ~ 1.24)	0.026

Model fit: Hosmer–Lemeshow test *p* = 0.618; Nagelkerke *R*^2^ = 0.26.

Model 1: no adjustment for other covariates; Model 2: adjusted for age, sex, and ethnicity; Model 3: adjusted for all covariates. *p*-values are based on Wald chi-square statistics from logistic regression.

#### 3.2.3 Interaction between BHEI index and extreme pessimistic thoughts

After adjusting for age, gender, ethnicity, BMI, total energy intake, alcohol consumption, smoking status, and physical activity, the interaction between extreme pessimism and BHEI status remained significantly associated with depression (*p* < 0.05). The results are summarized in Table 5. Compared to the non-pessimistic population with adequate BHEI scores, failure to meet the BHEI standard and extreme pessimism were each independently associated with increased odds of depression (*p* < 0.05). Notably, individuals with both substandard BHEI scores and extreme pessimism exhibited the highest risk of depression. Notably, the combination of low BHEI and extreme pessimism showed a markedly elevated depression risk (OR = 8.05), exceeding the effect of either factor alone.

**Table 5:** Interaction of the BHEI index with extreme pessimistic.

Variable	Phase × Interaction		Phase + Interaction	
	OR(95%CI)	*p*	OR(95%CI)	*p*
BHEI index compliance	1.47(1.20 ~ 1.66)	< 0.001	–	–
Extreme pessimistic thoughts	4.77(3.50 ~ 5.14)	< 0.001	–	–
BHEI non-compliance × Extreme pessimistic thoughts	1.21(1.03 ~ 1.62)	0.025	–	–
BHEI compliance + Non-extreme pessimistic thoughts	–	–	1	< 0.001
BHEI non-compliance + Non-extreme pessimistic thoughts	–	–	1.51(1.20 ~ 1.66)	< 0.001
BHEI compliance + Extreme pessimistic	–	–	4.49(3.50 ~ 5.14)	< 0.001
BHEI non-compliance + Extreme pessimistic thoughts	–	–	8.05(6.62 ~ 9.07)	< 0.001

Model fit: Hosmer–Lemeshow test *p* = 0.547; Nagelkerke *R*^2^ = 0.28.

*p*-values are based on Wald chi-square statistics from logistic regression.

## 4 Discussion

This study utilized NHANES data (2007–2018) to investigate the associations among the Balanced Healthy Eating Index (BHEI), extreme pessimism, depression, and their interactions. The results indicated that substandard dietary patterns (failing to meet the BHEI criteria) and extreme pessimism were each significantly associated with an increased risk of depression. Furthermore, interaction analyses revealed that participants simultaneously exhibiting substandard dietary patterns and extreme pessimism had the highest risk for depressive symptoms. This finding suggests a compounding effect, whereby poor diet and extreme cognition amplify one another’s impact on depressive symptoms. These findings are consistent with previous studies suggesting that low-quality diets increase the likelihood of mood disturbances, potentially due to nutritional deficiencies, pro-inflammatory processes, or metabolic dysregulation (16, 24). Finally, due to the cross-sectional design of NHANES, the possibility of reverse causality cannot be ruled out. For example, individuals with depression may adopt poorer dietary habits or experience increased pessimism as a consequence of their mental health status.

Our findings are consistent with a growing body of evidence highlighting the association between dietary quality and depression. For instance, several observational studies have reported that higher adherence to healthy dietary patterns, particularly the Mediterranean diet, is associated with a reduced risk of depressive symptoms and major depressive disorder (31, 32). Similarly, randomized controlled trials have shown that dietary interventions can lead to significant improvements in depressive symptoms, especially among young adults (33). A systematic review by Patsalos et al. (34) further emphasized the role of dietary improvement in managing depressive symptoms in overweight and obese individuals. In line with these findings, de Oliveira Meller et al. (35) reported that individuals with lower diet quality had a significantly higher likelihood of experiencing depressive episodes. These studies support the notion that dietary quality may influence mental health outcomes through multiple biological and psychosocial mechanisms (31). Collectively, our study adds to this literature by introducing the BHEI as a refined measure of diet quality and demonstrating its association with depressive risk and extreme pessimistic thinking in a large nationally representative population.

The finding that pessimistic thinking further increases the risk of depression highlights the multifactorial nature of mental health. Specifically, cognitive and emotional factors may exacerbate the negative effects of poor dietary habits on mood. Although key confounders—including age, sex, ethnicity, total energy intake, BMI, physical activity, alcohol consumption, and smoking status—were considered, residual confounding or reverse causation cannot be ruled out. For instance, individuals experiencing undiagnosed depressive symptoms might adopt unhealthy dietary habits, leading to a bidirectional relationship that complicates causal inference. Longitudinal and intervention studies are required to determine whether improving dietary quality can alleviate pessimistic thinking, or conversely, if enhancing cognitive functioning indirectly promotes healthier dietary behaviors. Future prospective studies could better clarify the temporal sequence of these factors, thereby informing targeted preventive strategies. One limitation is the operationalization of extreme pessimism using only a single PHQ-9 item, which may not fully capture the broader spectrum of negative cognitive styles and may lead to an underestimation of more moderate pessimistic tendencies.

Additionally, the reliance on self-reported dietary data in this study may have introduced measurement errors or recall bias. Future research should use prospective or longitudinal study designs, incorporate more comprehensive assessments of psychosocial stressors, and utilize objective dietary measures (e.g., biomarkers). Such refinements may help elucidate the mechanisms linking dietary quality, pessimistic cognition, and depressive outcomes. Given the cross-sectional nature of the NHANES data, the associations identified in this study do not imply causality.

## 5 Conclusion

This population-based cross-sectional study indicates that both suboptimal dietary quality (assessed by the Balanced Healthy Eating Index, BHEI) and extreme pessimistic thoughts are significantly associated with increased depression risk among adults aged 18–65 years. Moreover, the interaction between these two factors indicates a multiplicative effect on depressive symptoms, highlighting the importance of integrating nutritional and psychological interventions. Due to the inherent limitations of the cross-sectional design, this study cannot establish causality. Nevertheless, the findings highlight the potential importance of diet quality and extreme pessimism on depression risk. Further prospective and interventional studies are necessary to clarify temporal relationships and determine whether modifying dietary practices or pessimistic tendencies can effectively reduce depression incidence or severity.

## Data Availability

The original contributions presented in the study are included in the article/Supplementary material, further inquiries can be directed to the corresponding author.
