# Supplementary material for: Association between the Balanced Healthy Eating Index and depression and the mediating role of extreme pessimistic thoughts: an analysis from the health and nutrition database
Source: Front Nutr. 2025 Aug 19;12:1590171. doi: 10.3389/fnut.2025.1590171 (PMC12401681; doi:10.3389/fnut.2025.1590171)
Supplement: Supplementary file 1 [file Table_1.docx]

**Table S1.** B-HEI Weight details

| **Component** | **Original HEI Max** | **BHEI Weight (%)** | **Rationale** |
| --- | --- | --- | --- |
| Total Fruits | 5 | 6% | Anti-inflammatory; linked to improved mood |
| Whole Fruits | 5 | 7% | Rich in fiber and polyphenols |
| Total Vegetables | 5 | 6% | General antioxidant benefits |
| Greens and Beans | 5 | 6% | Folate content important for depression risk |
| Whole Grains | 10 | 7% | Lower risk of depressive symptoms |
| Dairy | 10 | 5% | Mixed evidence; moderate contribution |
| Total Protein Foods | 5 | 6% | Important for health but indirect link to depression |
| Seafood & Plant Proteins | 5 | 8% | Rich in omega-3s and tryptophan |
| Fatty Acid Ratio | 10 | 10% | Strong link to inflammation and depression |
| Refined Grains | 10 | 8% | High intake associated with mood disturbances |
| Sodium | 10 | 7% | Linked to vascular stress and mood instability |
| Added Sugars | 10 | 10% | Strongly associated with pessimism and depression |
| Saturated Fat | 10 | 4% | Weaker and inconsistent associations with depression |
| Total | 100 | 100% |  |
